# New conceptualization of suicidal behaviour

**DOI:** 10.1192/j.eurpsy.2025.36

**Published:** 2025-08-26

**Authors:** M. Nordentoft

**Affiliations:** 1 Copenhagen Research Center for Mental Health, Mental Health Center; 2 Department of Clinical Medicine, University of Copenhagen, Copenhagen, Denmark

## Abstract

**Abstract:**

Suicide is a huge public health problem with 700,000 deaths per year. United Nations are aiming to reduce suicide rates with 33 percent before 2030. Some countries have experienced declining trends, especially in Asia, partly due to restrictions in access to highly lethal pesticides. USA are facing increasing trends and in many western European countries there has been declining tendencies, but most recently rates have been rather stable.

Suicide preventive strategies have focused on universal prevention, thus intervention targeting the who population, selective prevention aiming to reduce risk in different high risk groups, and indicated prevention targeting people who are already having suicidal behavior. In most cases, suicidal acts are carried out within a short period of time, and in many cases without a long period of warning signals. In a way, suicidal acts resemble heart attacks or epileptic episodes more than other complications that often develop slowly and gradually. This makes the task of creating awareness programmes even more difficult.

However, a thorough mapping of risk groups and risk situations will enable us to plan a more targeted intervention. Thus, epidemiology and clinical research can play together.

The most important task is to identify those of immediate risk of suicide and provide treatment and support. There are four distinct risk groups with a very high suicide rate. These are 1) people sent home from psychiatric emergency room visits, 2) people recently discharged from psychiatric hospitalization 3) people who were hospitalized due to attempted suicide 4) people who have called life-line or other NGO-driven helplines because of suicidal thoughts. It can be helpful to evaluate the population attributable risk associated with different risk factors. The population attributable risk is an estimate of the proportion of the problem that could be avoided if the increased risk in a specific risk group could be reduced to the level of the general population. All these four groups have a very high risk of suicide and together they accounted for 25 - 50 percent of all suicides in Denmark, and the help they are offered are in most countries fragmented and not well organized. Coherent and assertive interventions are needed in order to reduce suicide rates. Interventions will need to involve monitoring persons in high-risk groups for longer periods.

All mental disorders are associated with increased risk of suicide, especially the first years after first hospital contact.

Safety plans have proven to be an effective tool for suicide prevention, and this concept will be presented

**Disclosure of Interest:**

None Declared

